# Pregnancy-Related Anxiety, Perceived Parental Self-Efficacy and the Influence of Parity and Age

**DOI:** 10.3390/ijerph17186709

**Published:** 2020-09-15

**Authors:** Robyn Brunton, Nicole Simpson, Rachel Dryer

**Affiliations:** 1 School of Psychology, Charles Sturt University, Bathurst 2795, Australia; nicolesimpson75@gmail.com; 2 School of Behavioural and Health Sciences, Australian Catholic University, Strathfield 2135, Australia; rachel.dryer@acu.edu.au

**Keywords:** pregnancy-related anxiety, maternal attitudes, prenatal attachment, parental expectations, parity, fear of childbirth

## Abstract

Pregnancy-related anxiety is contextualised by pregnancy and is a health concern for the mother and child. Perceived parental self-efficacy is associated with this anxiety and age and parity are identified as influential factors. This research, therefore, predicted that negative perceptions of parental self-efficacy would predict greater pregnancy-related anxiety, moderated by parity and age. Participants (*N* = 771) were recruited online and assessed for perceived parental self-efficacy, pregnancy-related anxiety, and demographics. Moderation models showed that the psychosocial and sociodemographic factors combined predicted up to 49% of the variance. Parental self-efficacy predicted anxiety in the areas of body image, worry about themselves, baby concerns, pregnancy acceptance, attitudes towards medical staff and childbirth, and avoidance. Parity predicted pregnancy-related anxiety both overall and in childbirth concerns, worry about self, baby concerns and attitudes towards childbirth. Age predicted baby concerns. There was a significant moderation effect for pregnancy acceptance indicating that primiparous women with low perceptions of parental self-efficacy are less accepting of their pregnancy. Results suggest that parity and parental self-efficacy may be risk factors for first-time mothers for pregnancy-related anxiety.

## 1. Introduction

Historically, pregnancy was regarded as a time of positive mental health [1] but it is now increasingly acknowledged that pregnancy may predispose some women to an increased vulnerability for anxiety [2] such as pregnancy-related anxiety. This distinct form of anxiety is contextualised by pregnancy and can occur at a severe level in as many as 22% of expectant mothers. Those with severe levels of pregnancy-related anxiety are placed at greater risk of adverse maternal, obstetric and neonatal outcomes [3,4,5]. Therefore, understanding the etiology of pregnancy-related anxiety and associated risk factors is essential to prenatal care.

Pregnancy-related anxiety is characterised by current and future concerns around pregnancy (e.g., childbirth, baby’s health, motherhood, [3]). These concerns may be heightened in certain stages of pregnancy with cross sectional studies identifying a U-shaped course with early and late pregnancy times of higher concern [5]. Several studies have also distinguished pregnancy-related anxiety from general, trait or state anxiety, depression and anxiety disorders diagnosed according to the Diagnostic and Statistical Manual of Mental Disorders [6,7,8,9] confirming its distinct status [4].

Pregnancy-related anxiety is not just a health concern for expectant mothers; the effect of this anxiety may also extend to the prenatal environment. Consistent with the Fetal Programming Hypothesis, the rapid development of the fetus increases its susceptibility to environmental influences. These influences may come from the associated physiological changes linked to increased anxiety, and at the critical stages of development may result in permanent alterations to physical and metabolic functions [10,11]. This may explain why pregnancy-related anxiety is consistently and robustly associated with deleterious outcomes such as preterm birth [7,12,13,14], developmental delays in children [15,16] and adverse childbirth and labour outcomes ([17,18,19], i.e., prolonged labour, more pain relief/sedation, [20]). These associations are less consistent or not evident for general anxiety or anxiety disorders (see [21] for a review). Given this, pregnancy-related anxiety is emerging as “a potent maternal risk factor” [3].

Sociodemographic variables such as age and parity have been identified as either being predictive of or associated with pregnancy-related anxiety. Madhavanprabhakaran et al. [5] noted that younger primiparous women (18–20 years) presented higher levels of pregnancy-related anxiety (using a 40-item pregnancy-specific anxiety scale assessing anxiety about being pregnant, childbirth, breastfeeding and newborn care) in comparison to older multiparous women (21–35 years). Indeed, childbirth anxieties were greater for the first-time mothers (than multiparous women) in later pregnancy, with fear of childbirth significantly higher for these women. Arch [22] noted that parity predicted pregnancy-related anxiety generally, and fear of giving birth specifically, such that having previous children predicted less fear. Qualitative analysis has shown that women with no previous pregnancies have greater labour pain fears, they fear problems developing during childbirth, and have concerns about attitudes of medical staff; consistent with key dimensions of pregnancy-related anxiety [23].

Conversely, others have not found differences between primiparous and multiparous women in their anxiety levels during pregnancy [24,25,26,27]. However, these studies assessed women in mid-pregnancy (as stated above, anxiety in pregnancy may follow a U-shaped curve) or used general anxiety measures which may have limited their findings. To date, only a few studies have examined parity and pregnancy-related anxiety, however, there are major limitations in many of these studies that include the use of measures that lack psychometric evidence or lack the breadth to fully examine the multidimensionality of this anxiety. The need therefore exists to examine the influence of parity on pregnancy-related anxiety using a comprehensive and psychometrically sound measure.

In a study that utilised a comprehensive measure of pregnancy-related anxiety, age was found to differentiate on two dimensions of pregnancy-related anxiety which are body image concerns and baby concerns. Younger women (18–25) had heightened concerns in both areas in comparison to older women (26–43, [28]). Similarly, Glazier and colleagues [27] found that state anxiety in pregnancy was inversely related to age (*r* = −0.17) in a large sample of women in early pregnancy (*N* = 2052, *M_age_* = 30.7, *SD* = 4.5). Gurung et al. [25] also noted in a younger sample (*N* = 453, *M_age_* = 28, *SD* = 1.58) that age was moderately correlated with attitudes towards pregnancy (*r* = 0.19) with older women having more positive attitudes. Moreover, after controlling for early prenatal anxiety, age was the only significant predictor of late prenatal anxiety with older women reporting less anxiety over time than other women (β = −0.12). In contrast to these findings, Arch [22] did not find age (*N* = 311, *M*_age_ = 27.47, *SD* = 5.97) to significantly predict pregnancy-related anxiety. However, their findings may have been limited by only examining multiparous women and assessing only three aspects of pregnancy-related anxiety.

Both parity and age have also been linked to perceived parental self-efficacy. Self-efficacy is a cognitive process whereby a person evaluates their capabilities to cope with or master different situations. In other words, it is the strength of one’s convictions about their effectiveness [29]. Perceived parental self-efficacy, therefore, is the evaluation of future parenting abilities. Razurel, Kaiser, Antonietti, Epiney, and Sellenet’s [30] longitudinal study examined 235 primiparous women aged 21–43 and the relationship between their perceived parental self-efficacy and stress, depression and anxiety. In this study perinatal stress was linked to perceived parental self-efficacy, such that the more significant the stress the more ineffective women felt about the motherhood role. Wernand and colleagues [31] examined parental self-efficacy and depression and anxiety in 533 primiparous women, finding that greater psychopathology was related to less self-efficacy in all pregnancy trimesters. Mohammad, Gamble and Creedy [32] examined both primiparous and multiparous women (*N* = 353) in late pregnancy noting that a perceived lack of maternal self-efficacy was a probable factor in antenatal depression. While these studies point to an association between perceived parental self-efficacy and psychopathology, most of the studies have assessed self-efficacy postpartum or have examined general self-efficacy and not parental self-efficacy. Further, many existing studies examined nulliparous women exclusively which limits knowledge on the influence of parity. To the best of our knowledge, no one has yet considered the influence of parity or age on the relationship between perceived parental self-efficacy and pregnancy-related anxiety.

Lastly, the trait of neuroticism is a potentially confounding variable when assessing anxiety measures [33]. Neuroticism is related to the vulnerability or predisposition to psychological distress and is often conveyed and measured as anxiety [33]. Given that van Bussel, et al. [34] found higher neuroticism scores consistently predicted greater anxiety, neuroticism needs to be controlled for in all analyses. However, none of the reviewed studies controlled for neuroticism in examining the association between age, parity and pregnancy-related anxiety, or in examining the relationship between parental self-efficacy and pregnancy-related anxiety.

This research, therefore, examined the influence of parity and age on perceived parental self-efficacy and pregnancy-related anxiety (controlling for neuroticism). It was predicted that a negative perception of parental self-efficacy would predict higher levels of pregnancy-related anxiety with the relationship moderated by parity and age. In other words, parity and age were expected to influence the strength of the relationship between parental self-efficacy and pregnancy-related anxiety, such that we would expect this relationship to be weaker in multiparous women in comparison to nulliparous women. Similarly, based on the findings of previous research, we would expect the relationship between perceived parenting self-efficacy and pregnancy-related anxiety to be weaker in older women in comparison to younger women.

## 2. Materials and Methods

### 2.1. Participants

Participants were eligible for the study if they were over 18 years of age and pregnant. The study commenced after institutional ethics approval (H19120) with 1210 women initially recruited. Of these, 246 self-reported a high-risk pregnancy (e.g., has a medical professional … or do you believe you are high-risk?) and were excluded as a requirements of ethics approval. A further 193 did not record any responses on the key measures as they exited the survey right at the beginning and were thus deleted. Given that these participants had no recorded data, we were unable to assess if they differed from the other participants. The final sample consisted of 771 pregnant women aged 18–41 (*M* = 26.44, *SD* = 5.36) who were predominantly Australian born, partnered, employed and had at least a high school education. Most women were in mid-pregnancy (*M*_gestation_ = 22.58 weeks, *SD* = 9.42) with just over 50% reporting they were first time mothers (see Table 1). The sample was a good representation of pregnant women in Australia, with the age range consistent with that of childbearing women [35].

### 2.2. Materials

The online survey consisted of three scales and demographic information. To avoid potential effects of priming, the scales were presented in the order below. After completion, participants were fully debriefed and informed of the study aims.

#### 2.2.1. Prenatal Parental Expectations Survey-Revised (PPES-R)

The 25-item PPES-R [36] is a measure of perceived parental self-efficacy validated for antenatal use (permission to use the scale was granted by Dr Reece). Items include “I can tell when my baby is sick” rated on a scale of 0 (cannot do) to 10 (certainly can do); higher scores indicate greater perceived parental self-efficacy. Convergent and predictive validity are shown by correlations with similar measures (i.e., the Confidence in Parenting subscale of the Postpartum Self Evaluation Questionnaire and the Perceived Stress Scale, *rs* = 0.40–0.75). The full scale has excellent internal consistency reliability prenatally (α = 0.92). The scale does lack evidence of internal validity and to the best of our knowledge, this is yet to be examined. Therefore, this study also examined the scale’s dimensionality.

#### 2.2.2. Pregnancy-Related Anxiety Scale (PrAS)

The PrAS [28] is a 32-item measure of pregnancy-related anxiety. The eight subscales assess dimensions of pregnancy-related anxiety (i.e., concerns about childbirth, the unborn baby and body image, attitudes towards childbirth and medical staff, worry about themselves, acceptance of pregnancy and avoidance). Responses are rated from 1 (not at all) to 4 (always) with higher scores indicating increased levels of pregnancy-related anxiety. The scale has good psychometric properties with evidence of content, concurrent, predictive and internal validity (see [28]) and excellent internal consistency reliability for the full scale (α = 0.92) and moderately-high to excellent reliability for the subscales ranging from α = 0.84 to 0.95 [37]. Note that for this study the word ‘husband’ was replaced with ‘partner’ for inclusiveness.

#### 2.2.3. International Personality Item Pool- Neuroticism Scale (IPIP-N)

The 10-item IPIP-N [38] assesses neuroticism with items such as “I often feel blue” scored on a scale from 1 (very inaccurate) to 5 (very accurate). Higher scores indicate greater neuroticism. The IPIP-N has moderately-high internal consistency reliability ranging from α = 0.83 to 0.87 [39], consistent with this study (α = 0.82). Concurrent validity has been demonstrated by high correlations (*r* = −0.84) with the NEO Five-Factor Inventory [40].

#### 2.2.4. Demographics

Demographic information included age, gestation, marital status, education level, employment status and country of birth. Parity was assessed as primiparous (never previously pregnant) and multiparous (only successful previous pregnancies). Women who had only previous unsuccessful pregnancies (e.g., previous miscarriages/stillbirths) were excluded from the analyses to reduce confounding.

### 2.3. Procedure

Recruitment was through a dedicated Facebook page with the survey administered via the Qualtrics platform. Online recruitment provided convenience and the ability to reach many participants with research showing that online responses do not differ significantly from traditional paper and pencil methods [41]. However, limitations of this method include a bias towards younger participants who have internet access/social media [42]. However, given that the study targeted pregnant Australian women (aged 18–45), this bias was not thought to limit this study. All survey data were anonymous, and participants had a chance to win one of two AUD 50 gift cards to incentivise recruitment. Participants were first provided with the study’s information statement that warned of possible distressing content and provided support services information. Proceeding to the survey indicated informed consent.

### 2.4. Statistical Analyses

Data were analysed using the IBM (New York, NY, USA) Statistical Package for the Social Sciences (v 25). Bootstrap resampling with bias-corrected and adjusted confidence intervals was used in all analyses. Cronbach’s alpha determined internal consistency reliability and described according to Murphy and Davidshofer’s [43] conventions. Pearson’s correlations were described using Sattler’s [44] conventions. Pairwise analysis was used for all analyses.

**Figure 1:**
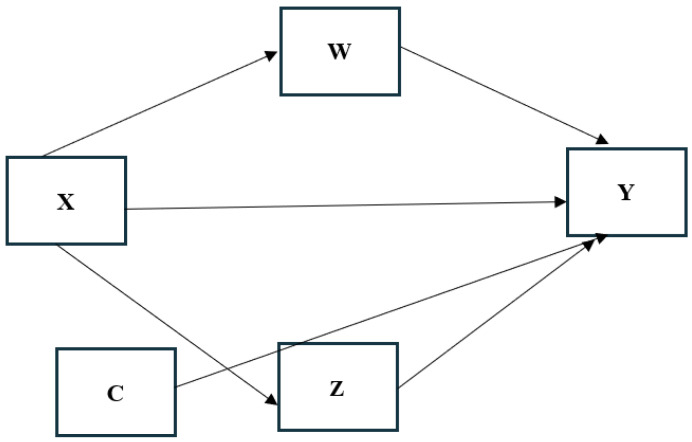
Moderation model.

Moderation analyses used Hayes’ [45] PROCESS (v3.4). All assumptions (i.e., the ratio of cases/predictors, linearity and homoscedasticity of residuals) were met. Multivariate outliers and multicollinearity were detected using Mahalanobis distance and tolerance and variance inflation factor statistics, respectively [46]. Nine moderation models examined perceived parental self-efficacy (X) as a predictor of pregnancy-related anxiety (Y) moderated by parity (W) and age (Z). Any covariates identified in the correlational analyses were controlled in the analyses (C). The model is depicted in Figure 1.

Multivariate analysis of variance (MANOVA) compared pregnancy-related anxiety scores and perceived parental self-efficacy between primiparous and multiparous women and two age groups consistent with previous research (e.g., [28]); 18–25 and 26 + with any identified covariates controlled for and 5000 bootstrap resamples used.

## 3. Results

Table 2 presents descriptive statistics for all dependent variables. Using a PrAS cut-off of 75.5 [40], participants were not considered to score at a clinical level of anxiety.

### 3.1. Preliminary Analyses

#### 3.1.1. Pearson’s Correlations

Correlations between all variables are shown in Table 3. There were small negative correlations between the PPES-R and the PrAS (both the full-scale and subscales) indicating that less perceived parental self-efficacy was associated with greater anxieties about pregnancy. The small associations between age and the PrAS and age and childbirth concerns, body image concerns, worry about themselves and attitudes towards medical staff indicate that as age increased these areas of concern decreased. Parity was related (small) to the PrAS overall and childbirth concerns, baby concerns, acceptance of pregnancy and attitudes towards childbirth, indicating that primiparous women have greater anxieties in these areas and overall, whereas multiparous women have less anxiety. There were small to large correlations between the PrAS, its subscales and the PPES-R with neuroticism and correlations between some PrAS subscales and education and gestation. These variables were therefore controlled for in all analyses.

#### 3.1.2. Exploratory Factor Analysis on the PPES-R

Factor analysis was undertaken using Principal Axis Factoring with oblique rotation (Promax) given that the underlying factors were likely correlated [47]. Assumptions of independence, item to sample size ratio, and normality were met. Data were confirmed suitable with most correlations > 0.30 and no multicollinearity detected [46]. The Kaiser–Meyer–Olkin (KMO) measure of sampling adequacy (KMO = 0.96), Bartlett’s test of sphericity (approx. Chi-square = 11,001.41, df = 300, *p* < 0.0001) and anti-image matrices further confirmed suitability.

Three factors explained 63.86% of the variance with the scree plot further confirming this factor structure. Table 4 shows the factor loadings and explained variance for each subscale. Examination of the items within each factor identified them as Practical Care, Emotional Support, and Knowing and Understanding. Five cross-loaded items with differences in factor loadings of <0.30 were individually checked and retained on the most relevant factor. For example, “I will be able to keep my baby from crying” loaded on both factor 1 and 3 but was retained on factor 3 as it was more relevant to knowing and understanding than practical care.

Items were further assessed for internal consistency reliability. Cronbach’s alpha for all subscales was excellent and no items, if removed, would improve the subscale reliability. The factor correlation matrix confirmed the factors were correlated but none exceeded *r* = 0.80 indicating that they were tapping into distinct areas of parental self-efficacy [48]. Table 5 shows the internal consistency reliability and inter-factor correlations.

Given the multidimensionality of the PPES-R, we further examined the correlations between these factors and all variables. As shown in Table 6, all PPES-R subscales had small negative relationships with the PrAS and its subscales indicating that less self-efficacy in all three areas was related to greater pregnancy-related anxiety, both overall and in all dimensions. Age was negatively associated with all three subscales. Parity had a small positive relationship with Practical Care, indicating that a first-time mother may have lower self-efficacy for the practical care of their baby.

#### 3.1.3. Moderation Analyses

Nine moderation models (i.e., one for each PrAS subscale and the total PrAS score) assessed if perceived parental self-efficacy predicted pregnancy-related anxiety and whether this was moderated by parity and age. Neuroticism, gestation and education were controlled for in the analyses. Given the PPES-R subscales all correlated similarly with the PrAS, moderation was completed on the full-scale PPES-R only. No multivariate outliers were detected using Mahalanobis distance (chi-square critical value = 13.82) and all data assumptions were met.

As reported in Table 7, all moderation models were statistically significant except for the Avoidance subscale. For the remaining eight models the psychosocial and sociodemographic factors combined predicted 41% of the variance in the overall model and between 11 and 49% in the subscale models. For the PrAS, body image concerns, worry about themselves, baby concerns, acceptance of pregnancy, attitudes towards medical staff, attitudes towards childbirth and avoidance; perceived parental self-efficacy significantly predicted pregnancy-related anxiety in these areas. However, perceived parental self-efficacy did not significantly predict childbirth concerns. Parity predicted pregnancy-related anxiety for the PrAS and childbirth concerns, worry about self, baby concerns and attitudes towards childbirth. Age was a significant predictor for baby concerns. There was a significant moderation effect for acceptance of pregnancy. Perceived parental self-efficacy and parity interacted suggesting that primiparous women with low parental self-efficacy are less accepting of their pregnancy.

**Figure 2:**
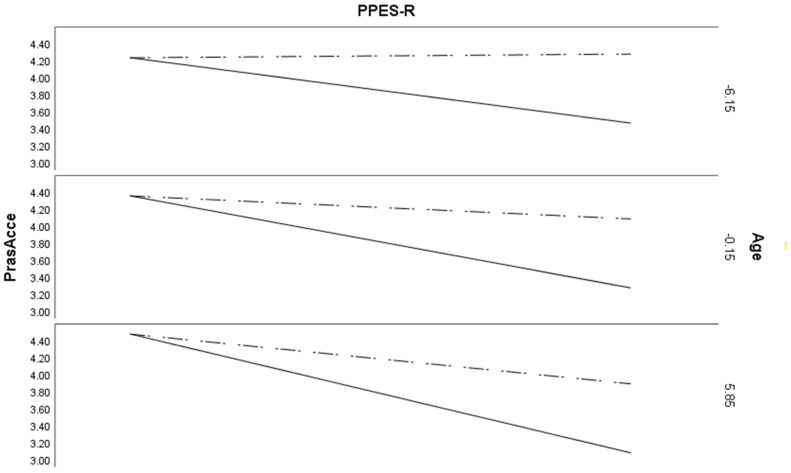
Acceptance of pregnancy, moderation effects. The dashed line represents multiparous women and the solid line represents primiparous women.

Figure 2 shows the regression of the PPES-R on PrAS acceptance when age is low (−6.15), centered (−0.15) and high (5.85) for both primiparous and multiparous women. Note that age was mean centered as zero is not a meaningful age in this analysis.

#### 3.1.4. MANOVA

Two MANOVAs compared primiparous and multiparous women and younger and older age groups (18–25 years and 26 years+) on the full-scale PrAS and PPES-R and their subscales. For both MANOVAs, the effects of neuroticism, education and gestation were controlled, and the assumptions of normality and linearity were supported by examination of the Shapiro–Wilk statistic, histograms and scatterplots.

The first MANOVA examined group differences on the full-scale PrAS and PPES-R. Multicollinearity and multivariate outliers were not problematic based on low correlations between the PrAS and PPES-R and Mahalanobis distance below the critical value of 13.816 (assessed for both independent variables). The assumption of homogeneity of variances and covariance were met by the non-significant Levene’s test (PrAS, *F*(3, 352) = 0.57, *p* = 0.63, PPES-R, *F*(3, 352) = 2.35, *p* = 0.07) and Box’s M, *p* = 0.32. After controlling for covariates, there was a significant between subject effect for parity on the PrAS *F*(1349) = 7.23, *p* = 0.008, partial *η^2^* = 0.02 but no significant effect for age *F*(1349) = 0.17, *p* = 0.68 and no significant interaction between parity and age *F*(1356) = 0.10, *p* = 0.75. There were no significant effects for the PPES-R for either parity, PrAS *F*(1349) = 2.08, *p* = 0.15, or age PrAS *F*(1349) = 1.48, *p* = 0.23. Post-hoc testing revealed that primiparous women had greater pregnancy-related anxiety (*N* = 229, *M* = 64.49, *SE* = 0.89) than multiparous women (*N* = 127, *M* = 60.37, *SE* = 1.24) with the pairwise comparison significant at *p* = 0.008.

The second MANOVA examined group differences for all PrAS and PPES-R subscales. There was some skewness in the distribution of some subscales however bootstrap resampling was used. Eight multivariate outliers were identified as they exceeded the critical Mahalanobis distance value of 31.26, *p* < 0.001 (parity = 39.59, age group = 40.73), with the cases excluded leaving a final sample of, *N_primiparous_* = 225, *N_multiparous_* = 125, *N_18–25 years_* = 171, *N_26 years+_* = 179. The assumption of homogeneity of variances and covariance were met by the non-significant Levene’s test for all subscales except the PrAS attitudes towards childbirth (*p* = 0.03), and the PPES-R subscales, practical care (*p* = 0.01) and emotional support (*p* = 0.01). This was not considered problematic as all subscales were assessed with a stricter Bonferroni adjusted alpha level of *p* <.0004 [46]. Box’s M (*p* = 0.05) indicated that the assumption of equality of covariances was not violated using the alpha *p* < 0.001 [46].

After controlling for the covariates, there was a significant between-subject effect for parity and childbirth concerns and attitudes towards childbirth. There was no significant effect for age group or any age group X parity interaction. The results for parity are reported in Table 8 with the full MANOVA results provided as supplementary data (Table S1: PrAS and PPES-R, and Table S2: PrAS subscales and PPES-R subscales).

## 4. Discussion

The current study extends knowledge on the factors that increase vulnerability for pregnancy-related anxiety through the examination of perceived parental self-efficacy, parity and age. As expected, an expectant mother’s negative perceptions of her parental self-efficacy predicted greater pregnancy-related anxiety. Lower levels of parental self-efficacy also predicted greater body image concerns, worry about themselves, baby concerns, avoidance, attitudes towards medical staff and attitudes towards childbirth. However, apart from the dimension of acceptance of pregnancy, parity or age did not moderate any of these relationships. For pregnancy acceptance, primiparous women with a low perception of parental self-efficacy were less accepting of their pregnancy. Parity also independently predicted pregnancy-related anxiety both overall and in the dimensions of baby concerns, childbirth concerns, attitudes towards childbirth and worry about themselves. Increased age independently predicted pregnancy-related anxiety in the area of baby concerns. Therefore, our predictions were partially supported.

While these findings are consistent with those of Razurel, et al. [30] and Wernand, et al. [31] who found that less parental self-efficacy predicted general anxiety in pregnancy, they extend knowledge by considering the more specific pregnancy-related anxiety. Further, this study delineates the results to understand the specific dimensions of pregnancy-related anxiety that perceived parental self-efficacy predicts. The finding that age and parity did not interact with parental self-efficacy was unexpected. However, it is worth noting that both age and parity did independently predict different aspects of pregnancy-related anxiety, consistent with previous research [5,28].

Both perceptions of parental self-efficacy and parity significantly and independently predicted pregnancy-related anxiety, overall. Indeed, the stronger predictor of the two was parity in that having no previous experience with pregnancy and childbirth predicted higher pregnancy-related anxiety scores, more so than poor perceptions of an expectant mother’s abilities in the area of parenthood. Parental self-efficacy and parity also independently predicted attitudes towards childbirth with parity again the stronger predictor. In the area of childbirth concerns, attitudes towards childbirth and attitudes towards medical staff; these were predicted by parity alone, which is consistent with this area of concern/attitudes related to the birth experience. Having previous children likely provides a protective factor for childbirth concerns with a positive previous experience increasing knowledge, expectations and self-efficacy for birthing [22]. Conversely, when childbirth is a first-time experience, fear of the unknown would likely be greater [49]. Additionally, the finding that parity is a stronger predictor than perceived parental self-efficacy is not surprising given that primiparous women have no experience with parenting and therefore would have limited knowledge to base their parental self-efficacy expectancies on.

Childbirth is a subjective and multidimensional experience influenced and impacted by various factors including fears for the baby, medical interventions and the actions of medical personnel [50,51]. Traumatic childbirth is thought to be experienced by as many as 48% of women and has consequences that may impact the care and the development of the child such as parenting distress, difficulties with mother–child interactions and forming a positive attachment (see [52] for a review). Therefore, knowing that parity may be a key factor in childbirth fears provides avenues for intervention. That is, the ‘fear of the unknown’ could be lessened for primiparous women through targeted information about childbirth, the childbirth environment and health-care personnel, key areas previously identified by primiparous women as contributing to childbirth fear [23].

Parental self-efficacy, parity and age all independently predicted baby concerns. While it was expected that poorer perceptions of parental self-efficacy and primiparity would be linked to increased concerns for the unborn baby’s welfare; increased age predicting greater concerns was unexpected. However, the increasing trend toward women delaying birth until over the age of 35, may offer an explanation as older women may be more likely to be first-time mothers [53]. Older women may also perceive higher pregnancy risks for both themselves and their babies which can manifest as having more baby concerns [53].

A key finding of this study is that poorer perceptions of parental self-efficacy predicts less pregnancy acceptance and this relationship was positively influenced by parity (i.e., increased parity decreased the strength of the relationship). Pregnancy acceptance is a key developmental task of pregnancy and the ability to see yourself as a mother is considered central to maternal psychological preparation for parenting [54,55]. Less acceptance of pregnancy is linked to postnatal parenting stress and poorer infant attachment [55,56,57] with women who feel more negatively towards their pregnancy known to seek less prenatal care and be at increased risk of poor neonatal outcomes [58,59]. Conversely, a woman’s positive attitude toward pregnancy and motherhood is linked to higher cognitive development scores in children at 2 years [54]. While these findings are consistent with others who have shown that feelings of uncertainty about pregnancy predict antenatal state anxiety, the literature around this topic is limited [24]. This current study, therefore, adds to our understanding of the topic and is one of the first to examine the role of parental self-efficacy, parity and pregnancy acceptance as a dimension of pregnancy-related anxiety.

Finally, multivariate analyses confirmed that nulliparous women have greater pregnancy-related anxiety compared to multiparous women. These increased scores for first-time mothers are consistent with the literature [22,26] but to the best of our knowledge, the comparison between primiparous and multiparous women has not previously been conducted using a comprehensive measure of pregnancy-related anxiety. Knowing that primiparous women are more likely to have higher pregnancy-related anxiety scores than women who have previously given birth, provides valuable insight into the care and support needed for first-time mothers.

The non-significant difference in pregnancy-related anxiety scores by age group is inconsistent with previous research for pregnancy-related anxiety [28]. The non-significant findings are however consistent with studies on advanced maternal age (i.e., 35 years +) with no differences in pregnancy-related anxiety scores between younger women (20–29) and older women (35+) [53].

Despite the addition to the research this study has provided, several limitations must be acknowledged. First, the study is cross-sectional which limits information on causality. Future studies using longitudinal data are needed. Sample size also limited the findings with some modest moderation results and analyses on the subscales not possible due to the limited sample when stratified. Future studies with larger samples are needed to provide more power. Another limitation is the reliance on women’s reports for all the measures used. While the use of more objective measures is often used in psychological research to address the issue of common variance, this is difficult to do due to the absence of more objective indicators of pregnancy-related anxiety. Finally, we acknowledge that there are many factors that may influence a woman’s pregnancy-related anxiety (e.g., childhood trauma, unplanned pregnancy, social support). While these were not assessed in this study, future studies would benefit from considering these potential covariates.

## 5. Conclusions

The findings of this study contribute to the accumulating evidence for potential antecedents for pregnancy-related anxiety providing increased opportunity for prevention, education and intervention. Interventions aimed at identifying and reducing anxieties about pregnancy would be advantageous to the health and wellbeing of both mother and child. The findings also relate to Beck’s (1985) original theory on anxiety that it often stems from an overestimation of a threat and/or an under-estimation of coping skills or resources concerning that perceived threat. This is particularly true for the findings of parental self-efficacy, being the belief in one’s abilities to cope with a situation. Low expectations of parental self-efficacy could increase pregnancy-related anxiety which is an important insight from a prevention and education perspective, particularly for primiparous women. The differences between primiparous and multiparous women offer fresh insight into the potential elevated risk for first-time mothers to present with higher levels of pregnancy-related anxiety suggesting that parity is a key sociodemographic variable in antenatal screening.

## Figures and Tables

**Table 1:** Participant Demographics.

	Number	Percentage (%)
Gestation ^a^		
1st trimester (≤13 weeks)	96	20.16%
2nd trimester (14–26 weeks)	206	43.28%
3rd trimester (>27 weeks)	174	36.56%
Parity ^a^		
No previous pregnancies	246	51.68%
Only successful previous pregnancies	141	29.62%
Marital Status ^b^		
Partnered	433	84.74%
Single/Divorced/Widowed	78	15.26%
Education ^b^		
Low	196	38.36%
Medium	184	36.00%
High	131	25.64%
Employment ^b^		
Employed/Self-employed	281	55.00%
Homemaker	186	36.40%
Unemployed/Student	44	8.60%
Country of birth ^b^		
Australia	427	83.36%
India	17	3.33%
United Kingdom	14	2.75%
New Zealand	14	2.75%
Other	39	7.63%

Missing data, a = 295, b = 260. Education: low = high school, medium = certificate/diploma, high = undergraduate degree or higher. Parity excludes 384 women who indicated previous unsuccessful pregnancies.

**Table 2:** Descriptive statistics for scales and subscales.

	Mean	SD	N	ICR
Pregnancy-related Anxiety Scale	63.03	16.45	515	0.92
Childbirth Concerns	2.12	0.82	537	0.87
Body Image Concerns	2.30	0.92	537	0.91
Attitudes towards Childbirth	2.55	0.86	537	0.85
Worry about themselves	1.81	0.64	515	0.85
Baby Concerns	1.95	0.89	515	0.92
Acceptance of Pregnancy	1.31	0.53	515	0.75
Avoidance	1.47	0.70	515	0.84
Attitudes towards Medical Staff	2.00	0.92	515	0.91
PPES-R	8.31	1.67	573	0.96

PPES-R = Prenatal Parent Expectations Survey-Revised, theoretical range 0–25. Mean scores reported for all subscales and the PPES-R. ICR = internal consistency reliability, reported as Cronbach’s alpha.

**Table 3:** Correlations between the pregnancy-related anxiety scale (PrAS), PPES-R, international personality item pool (IPIP) and demographic variables.

	PrAS	CB	BI	Worry	BC	Accept	Avoid	Med	Att	IPIP	PPES-R	Age	Gest	Parity
IPIP	0.57 **	0.27 **	0.55 **	0.66 **	0.32 **	0.23 **	0.11 *	0.36 **	0.24 **					
PPES-R	−0.36 **	−0.17 **	−0.21 **	−0.32 **	−0.26 **	−0.25 **	−0.13 *	−0.16 **	−0.37 **	−0.17 **				
Age	−0.06	−0.02	−0.07	−0.14 **	0.13 *	0.05	−0.04	−0.12 **	−0.03	−0.24 **	−0.13 *			
Gest	−0.06	−0.01	0.09	−0.06	−0.16 **	−0.07	−0.01	−0.12*	−0.13 *	−0.01 *	0.01	0.08		
Parity	−0.12 *	−0.19 **	0.03	−0.09	−0.10 **	0.10 *	−0.05	−0.04	−0.23 **	0.02	0.07	0.23 **	0.01	
Educ	−0.05	0.04	−0.11 *	−0.06	0.07	0.05	−0.03	−0.12 *	−0.07	−0.20 **	−0.04	0.48 **	0.05	−0.04

PrAS = Pregnancy-related anxiety scale, CB = childbirth concerns, BI = body image concerns, Worry = worry about themselves, BC = baby concerns, Accept = acceptance of pregnancy, Avoid = avoidance, Med = attitudes towards medical staff, Att = attitudes towards childbirth. IPIP = international personality item pool neuroticism scale, PPES-R = prenatal parent expectations survey-revised, Gest = gestation, Edu = education, ranging from low to high. Significance denoted as * *p* < 0.05, ** *p* < 0.01 (2-tailed). Correlations based on 2000 bootstrap resamples. *N* = 474.

**Table 4:** Factor loadings for PPES-R.

Item	Practical Care	Emotional Support	Knowing and Understanding
PPES6	0.83		
PPES4	0.83		
PPES3	0.83		
PPES5	0.79		
PPES7	0.64		
PPES2	0.64		
PPES1	0.61		
PPES8	0.57		
PPES9	0.51		
PPES10	0.42		
PPES13	0.39		
PPES22		0.93	
PPES25		0.86	
PPES18		0.81	
PPES16		0.72	
PPES24		0.65	
PPES15		0.63	
PPES14		0.59	
PPES19		0.41	
PPES12		0.36	
PPES21			0.71
PPES20			0.66
PPES23			0.38
PPES17			0.35
PPES11			0.31
Variance Explained	53.50%	6.29%	4.07%

Item numbers relate to the survey item numbers which were sourced directly from Dr Susan Reece who consented to the use of the scale in this study.

**Table 5:** Factor correlation matrix and reliability.

Subscales	Practical Care (α = 0.94)	Emotional Support
Emotional Support (α = 0.90)	0.72	
Knowing and Understanding (α = 0.86)	0.65	0.64

Internal consistency reliability (Cronbach’s alpha) is shown in parentheses.

**Table 6:** Correlations between PrAS, IPIP and demographic variables and the PPES-R subscales.

	PrAS	CB	BI	Worry	BC	Accept	Avoid	Med	Att	IPIP	Age	Gest	Parity	Edu
Practical	−0.30 **	−0.14 **	−0.17 **	−0.29 **	−0.27 **	−0.20 **	−0.10 *	−0.09	−0.33 **	−0.14 **	−0.12 *	0.01	0.12 *	−0.05
Emotional	−0.36 **	−0.18 **	−0.21 **	−0.35 **	−0.24 **	−0.32 **	−0.16 **	−0.18 **	−0.28 **	−0.20 **	−0.14 **	0.03	−0.01	−0.05
Knowing	−0.29 **	−0.13 *	−0.19 **	−0.24 **	−0.21 **	−0.21 **	−0.04	−0.11 *	−0.38 **	−0.12 *	−0.18 *	−0.06	0.03	−0.04

PrAS = Pregnancy-related anxiety scale, CB = childbirth concerns, BI = body image concerns, Worry = worry about themselves, BC = baby concerns, Accept = acceptance of pregnancy, Avoid = avoidance, Med = attitudes towards medical staff, Att = attitudes towards childbirth, IPIP = international personality item pool neuroticism scale, Gest = gestation, Edu = education, ranging from low to high. Significance denoted as * *p* < 0.05, ** *p* < 0.01 (2-tailed). Correlations based on 2000 bootstrap resamples. *N* = 474.

**Table 7:** Total and indirect effect of perceived parental self-efficacy on pregnancy-related anxiety.

	R^2^	β	SE β	CI β (95%)	*t*
PrAS	0.41	*F* (8347) = 31.57 ****			
PPES-R		−2.72 ****	0.55	−3.80–−1.64	−4.95
Parity		−3.93**	1.52	−6.91–−0.95	−2.59
PPES-RxParity		1.04	0.83	−0.59–2.67	1.26
Age		0.15	0.17	−0.19–0.49	0.89
PPES-RxAge		−0.10	0.07	−0.24–0.05	−1.29
Childbirth Concerns	0.11	*F* (8347) = 5.64 ****			
PPES-R		−0.25	0.21	−0.67–0.17	−1.19
Parity		−1.73 ***	0.55	−2.81–−0.65	−3.14
PPES-RxParity		−0.11	0.31	−0.73–0.50	−0.36
Age		0.03	0.06	−0.09–0.15	0.45
PPES-RxAge		0.01	0.02	−0.04–0.06	0.54
Body Image Concerns	0.33	*F* (8347) = 25.85 ****			
PPES-R		−0.36 *	0.16	−0.67–−0.05	−2.26
Parity		0.21	0.44	−0.65–1.08	0.49
PPES-RxParity		0.14	0.24	−0.22–0.61	0.58
Age		0.04	0.05	−0.46–0.13	0.95
PPES-RxAge		−0.03	0.02	−0.07–0.01	−1.26
Worry about Self	0.49	*F* (8347) = 40.02 ****			
PPES-R		−0.62 ****	0.12	−0.85–−0.38	−5.18
Parity		−0.66 *	0.33	−1.31–−0.01	−1.99
PPES-RxParity		0.32	0.20	−0.08–0.72	1.58
Age		−0.03	0.04	−0.10–0.04	−0.71
PPES-RxAge		−0.01	0.02	−0.04–0.03	−0.51
Baby Concerns	0.21	*F* (8347) = 12.22 ****			
PPES-R		−0.30 **	0.11	−0.51–−0.08	−2.74
Parity		−0.69 **	0.27	−1.20–−0.17	−2.61
PPES-RxParity		0.08	0.16	−0.23–0.40	0.52
Age		0.10 ***	0.03	0.04–0.15	3.44
PPES-RxAge		−0.01	0.01	−0.03–0.02	−0.30
Acceptance	0.15	*F* (8347) = 6.53 ****			
PPES-R		−0.32 ****	0.08	−0.49–−0.16	−3.87
Parity		0.41 *	0.19	0.04–0.79	2.15
PPES-RxParity		0.24 *	0.12	0.01–0.48	2.00
Age		−0.01	0.02	−0.05–0.03	−0.31
PPES-RxAge		−0.02	0.01	−0.04–0.01	−1.35
Avoidance	0.04	*F* (8347) = 1.65			
PPES-R		−0.20 *	0.10	−0.40–−0.01	−2.01
Parity		−0.14	0.26	−0.64–0.37	−0.53
PPES-RxParity		0.15	0.15	−0.15–0.44	0.96
Age		−0.03	0.03	−0.08–0.03	−0.95
PPES-RxAge		−0.03	0.02	−0.06–0.01	−1.79
Medical staff	0.15	*F* (8347) = 11.09 ****			
PPES-R		−0.22 *	0.10	−0.42–−0.02	−2.16
Parity		−0.17	0.30	−0.75–0.41	−0.57
PPES-RxParity		0.24	0.17	−0.10–0.57	1.40
Age		−0.01	0.04	−0.08–0.07	−0.13
PPES-RxAge		−0.01	0.01	−0.04–0.02	−0.86
Attitudes childbirth	0.21	*F* (8347) = 13.83 ****			
PPES-R		−0.44 ****	0.08	−0.60–−0.28	−5.26
Parity		−1.18 ****	0.29	−1.74–−0.61	−4.08
PPES-RxParity		−0.01	0.15	−0.30–0.28	−0.07
Age		0.05	0.03	−0.01–0.10	1.67
PPES-RxAge		−0.01	0.01	−0.03–0.01	−1.07

PPES-R and age were mean centered. Primiparous = 249, Multiparous = 138. Significance denoted as **** *p* < 0.0001, *** *p* < 0.001, ** *p* < 0.01, * *p* < 0.05. Model fit determined by Huber–White F statistic. PrAS = Pregnancy-related anxiety scale, Acceptance = acceptance of pregnancy, Medical staff = attitudes towards medical staff, Attitudes childbirth = attitudes towards childbirth, PPES-R = prenatal parent expectations survey-revised. Analysis based on 2000 bootstrap resamples.

**Table 8:** Tests of between-subjects effects for PrAS and PPES-R subscales and parity.

			Primiparous		Multiparous	
	F	η^2^	M (SE)	95% BCa	M (SE)	95% BCa
PrAS subscales						
Childbirth Concerns	9.80 *	0.03	2.25 (0.05)	2.15–2.36	1.97 (0.07)	1.82–2.11
Body Image	0.22	0.01	2.27 (0.05)	2.17–2.37	2.31 (0.07)	2.17–2.46
Attitudes Childbirth	17.20 *	0.05	2.68 (0.05)	2.58–2.79	2.30 (0.08)	2.15–2.44
Worry about Self	4.78	0.01	1.83 (0.03)	1.77–1.89	1.71 (0.05)	1.62–1.80
Baby Concerns	3.50	0.01	1.99 (0.05)	1.89–2.10	1.82 (0.07)	1.68–1.97
Acceptance	4.23	0.01	1.27 (0.03)	1.20–1.33	1.39 (0.05)	1.29–1.48
Avoidance	0.16	0.00	1.48 (0.05)	1.39–1.57	1.44 (0.06)	1.32–1.57
Attitudes Medical	0.05	0.00	2.01 (0.06)	1.89–2.12	1.98 (0.08)	1.82–2.14
PPES-R						
Practical Care	5.48	0.02	7.82 (0.12)	7.58–8.06	8.32 (0.17)	7.98–8.66
Emotional Support	0.01	0.00	9.02 (0.11)	8.80–9.24	9.01 (0.16)	8.70–9.32
Knowing	0.71	0.01	7.55 (0.12)	7.32–7.78	7.72 (0.16)	7.40–8.03

Significance denoted as * assessed at the Bonferroni adjusted value of *p* < 0.004. Attitudes Childbirth = a0ttitudes towards childbirth, Attitudes Medical = attitudes towards medical staff, Knowing = knowing and understanding. 95% BCa = 95% bias correct and adjusted confidence intervals. η^2^ = Partial Eta Squared, SE = Stand error. Analyses based on 5000 bootstrap resamples.

## Supplementary Materials

The following are available online at https://www.mdpi.com/1660-4601/17/18/6709/s1, Table S1. MANOVA: PrAS and PPES-R. Table S2. MANOVA: PrAS subscales and PPES-R subscales.

## References

[1] Spinelli M.G. (1997). Interpersonal psychotherapy for depressed antepartum women: A pilot study. Am. J. Psychiatry.

[2] Lee A.M., Lam S.K., Lau S.M.S.M., Chong C.S.Y., Chui H.W., Fong D.Y.T. (2007). Prevalence, course, and risk factors for antenatal anxiety and depression. J. Obstet. Gynecol..

[3] Dunkel-Schetter C., Tanner L. (2012). Anxiety, depression and stress in pregnancy: Implications for mothers, children, research and practice. Curr. Opin. Psychiatry.

[4] Bayrampour H., Ali E., McNeil D.A., Benzies K., MacQueen G., Tough S. (2015). Pregnancy-related anxiety: A concept analysis. Int. J. Nurs. Stud..

[5] Madhavanprabhakaran G., D’Souza M.S., Nairy K.S. (2015). Prevalence of pregnancy anxiety and associated factors. Int. J. Afr. Nurs. Sci..

[6] Huizink A., Mulder E., Robles de Medina P., Visser G.H.A., Buitelaar J. (2004). Is pregnancy anxiety a distinctive syndrome?. Early Hum. Dev..

[7] Blackmore E.R., Gustafsson H., Gilchrist M., Wyman C., O’Connor T.G. (2016). Pregnancy-related anxiety: Evidence of distinct clinical significance from a prospective longitudinal study. J. Affect. Disord..

[8] Brunton R.J., Dryer R., Saliba A., Kohlhoff J. (2018). Re-examining Pregnancy-related Anxiety: A replication study. Women Birth.

[9] Anderson C.M., Brunton R.J., Dryer R. (2019). Pregnancy-related anxiety: Re-examining its distinctiveness. Aust. Psychol..

[10] Kwon E.J., Kim Y.J. (2017). What is fetal programming? A lifetime health is under the control of in utero health. Obstet. Gynecol. Sci..

[11] Dunkel-Schetter C. (2011). Psychological science on pregnancy: Stress processes, biopsychosocial models, and emerging research issues. Annu. Rev. Psychol..

[12] Mancuso R.A., Dunkel-Schetter C., Rini C.M., Roesch S.C., Hobel C.J. (2004). Maternal prenatal anxiety and corticotropin-releasing hormone associated with timing of delivery. Psychosom. Med..

[13] Roesch S.C., Schetter C.D., Woo G., Hobel C.J. (2004). Modeling the types and timing of stress in pregnancy. Anxiety Stress Copin.

[14] Kramer M.S., Lydon J., Seguin L., Goulet L., Kahn S.R., McNamara H., Genest J., Dassa C., Chen M.F., Sharma S. (2009). Stress pathways to spontaneous preterm birth: The role of stressors, psychological distress, and stress hormones. Am. J. Epidemiol..

[15] Huizink A., Robles de Medina P., Mulder E., Visser G., Buitelaar J. (2003). Stress during pregnancy is associated with developmental outcome in infancy. J. Child Psychol. Psychiatry.

[16] Huizink A., Robles de Medina P., Mulder E., Visser G., Buitelaar J. (2002). Psychological measures of prenatal stress as predictors of infant temperament. J. Am. Acad. Child Psychiatry.

[17] Madhavanprabhakaran G., Kumar K., Ramasubramaniam S., Akintola A. (2013). Effects of pregnancy related anxiety on labour outcomes: A prospective cohort study. J. Res. Nurs. Midwifery.

[18] Rubertsson C., Hellström J., Cross M., Sydsjö G. (2014). Anxiety in early pregnancy: Prevalence and contributing factors. Arch. Women Mental. Health.

[19] Saisto T., Salmela-Aro K., Nurmi J.-E., Halmesmäki E. (2001). Psychosocial characteristics of women and their partners fearing vaginal childbirth. Brit. J. Obstet. Gynaecol..

[20] Koelewijn J.M., Sluijs A.M., Vrijkotte T.G. (2017). Possible relationship between general and pregnancy-related anxiety during the first half of pregnancy and the birth process: A prospective cohort study. BMJ Open.

[21] Alder J., Fink N., Bitzer J., Hosli I., Holzgreve W. (2007). Depression and anxiety during pregnancy: A risk factor for obstetric, fetal and neonatal outcome? A critical review of the literature. J. Mat. Fetal Neonatal Med..

[22] Arch J.J. (2013). Pregnancy-specific anxiety: Which women are highest and what are the alcohol-related risks?. Compr. Psychiatry.

[23] Sercekus P., Okumus H. (2009). Fears associated with childbirth among nulliparous women in Turkey. Midwifery.

[24] Akiki S., Avison W.R., Speechley K.N., Campbell M.K. (2016). Determinants of maternal antenatal state-anxiety in mid-pregnancy: Role of maternal feelings about the pregnancy. J. Affect. Disord..

[25] Gurung R.A.R., Dunkel-Schetter C., Collins N., Rini C.K., Hobel C.J. (2005). Psychosocial predictors of prenatal anxiety. J. Soc. Clin. Psychol..

[26] Teixeira C., Figueiredo B., Conde A., Pacheco A., Costa R. (2009). Anxiety and depression during pregnancy in women and men. J. Affect. Disord..

[27] Glazier R.H., Elgar F.J., Goel V., Holzapfel S. (2004). Stress, social support, and emotional distress in a community sample of pregnant women. J. Psychosom. Obst. Gynecol..

[28] Brunton R.J., Dryer R., Krageloh C., Saliba A., Kohlhoff J., Medvedev O. (2018). The Pregnancy-related Anxiety Scale: A validity examination using Rasch analysis. J. Affect. Disord..

[29] Bandura A. (1977). Self-efficacy: Toward a unifying theory of behavioral change. Psychol. Rev..

[30] Razurel C., Kaiser B., Antonietti J.-P., Epiney M., Sellenet C. (2017). Relationship between perceived perinatal stress and depressive symptoms, anxiety, and parental self-efficacy in primiparous mothers and the role of social support. Women Health.

[31] Wernand J.J., Kunseler F.C., Oosterman M., Beekman A.T., Schuengel C. (2014). Prenatal changes in parenting self-efficacy: Linkages with anxiety and depressive symptoms in primiparous women. Inf. Ment. Health J..

[32] Mohammad K., Gamble J., Creedy D. (2011). Prevalence and factors associated with the development of antenatal and postnatal depression among Jordanian women. Midwifery.

[33] Pluess M., Bolten M., Pirke K.-M., Hellhammer D. (2010). Maternal trait anxiety, emotional distress, and salivary cortisol in pregnancy. Biol. Psychol..

[34] Van Bussel J., Spitz B., Demyttenaere K. (2009). Anxiety in pregnant and postpartum women. An exploratory study of the role of maternal orientations. J. Affect. Disord..

[35] AIHW (2015). Australia’s Mothers and Babies 2013—in Brief.

[36] Reece S.M., Harkless G. (1998). Self-efficacy, stress, and parental adaptation: Applications to the care of childbearing families. J. Fam. Nurs..

[37] Brunton R.J., Dryer R., Saliba A., Kohlhoff J. (2019). The initial development of the Pregnancy-related anxiety Scale. Women Birth.

[38] Goldberg L.R., Johnson J.A., Eber H.W., Hogan R., Ashton M.C., Cloninger C.R., Gough H.G. (2006). The international personality item pool and the future of public-domain personality measures. J. Res. Pers..

[39] De Hoogh A.H.B., Den Hartof D.N. (2009). Neuroticism and locus of control as moderators of the relationships of charismatic and autocratic leadership with burnout. J. Appl. Psychol..

[40] Boyes M.E., French D.J. (2010). Neuroticism, stress, and coping in the context of an anagram-solving task. Pers. Individ. Differ..

[41] Weigold A., Weigold I.K., Russell E.J. (2013). Examination of the equivalence of self-report survey-based paper-and-pencil and internet data collection methods. Psychol. Methods.

[42] Bennetts S.K., Hokke S., Crawford S., Hackworth N.J., Leach L.S., Nguyen C., Nicholson J.M., Cooklin A.R. (2019). Using paid and free Facebook methods to recruit Australian parents to an online survey: An evaluation. J. Med. Internet Res..

[43] Murphy K.R., Davidshofer C.O. (2001). Psychological Testing: Principles and Applications.

[44] Sattler J.M. (2008). Assessment of Children: Cognitive Foundations.

[45] Hayes A.F. (2013). Introduction to Mediation, Moderation and Conditional Process Analysis: A Regression-Based Approach.

[46] Allen P., Bennett K., Heritage B. (2014). SPSS Statistics Version 22: A practical Guide.

[47] Field A. (2013). Discovering Statistics Using IBM SPSS Statistics.

[48] Mitchell M.L., Jolley J.M. (2007). Research Design Explained.

[49] Fisher C., Hauck Y., Fenwick J. (2006). How social context impacts on women’s fears of childbirth: A Western Australian example. Soc. Sci. Med..

[50] Sorenson D.S., Tschetter L. (2010). Prevalence of Negative Birth Perception, Disaffirmation, Perinatal Trauma Symptoms, and Depression Among Postpartum Women. Perspect. Psychiatry Care.

[51] Soet J.E., Gregory M.A., Brack A., DiIorio C. (2003). Prevalence and predictors of women’s experience of psychological trauma during childbirth. Birth.

[52] Simpson M., Catling C. (2016). Understanding psychological traumatic birth experiences: A literature review. Women Birth.

[53] Bayrampour H., Heaman M., Duncan K.A., Tough S. (2012). Comparison of Perception of Pregnancy Risk of Nulliparous Women of Advanced Maternal Age and Younger Age. J. Midwifery Women Health.

[54] Deave T. (2005). Associations between child development and women’s attitudes to pregnancy and motherhood. J. Reprod. Infant. Psychol..

[55] Hart R., McMahon C.A. (2006). Mood state and psychological adjustment to pregnancy. Arch. Women Mental Health.

[56] Ispa J.M., Sable M.R., Porter N., Csizmadia A. (2007). Pregnancy acceptance, parenting stress, and toddler attachment in low-income black families. J. Marriage Fam..

[57] Lederman R.P., Lederman E., Work B.A., McCann D.S. (1979). Relationship of psychological factors in pregnancy to progress in labor. Nurs. Res..

[58] Hulsey T.M. (2001). Association Between Early Prenatal Care and Mother’s Intention of and Desire for the Pregnancy. J. Obstet. Gyn. Neonatal Nurs..

[59] Keeley R.D., Birchard A., Dickinson P., Steiner J., Dickinson L.M., Rymer S., Palmer B., Derback T., Kempe A. (2004). Parental attitudes about a pregnancy predict birth weight in a low-income population. Ann. Fam. Med..

