# Supplementary material for: Pregnancy-Related Anxiety, Perceived Parental Self-Efficacy and the Influence of Parity and Age
Source: Int J Environ Res Public Health. 2020 Sep 15;17(18):6709. doi: 10.3390/ijerph17186709 (PMC7557851; doi:10.3390/ijerph17186709)
Supplement: Supplementary file 1 [file ijerph-17-06709-s001.pdf]

Table S1. MANOVA: PrAS and PPES-R

Tests of Between-Subjects Effects

Source	Dependent Variable	Type III Sum of Squares	df	Mean Square	F
Corrected Model	PrAS	32832.693[a]	6	5472.116	31.076
	PPES-R	48.023[b]	6	8.004	2.957
Intercept	PrAS	5180.373	1	5180.373	29.419
	PPES-R	1041.254	1	1041.254	384.631
IPIP	PrAS	30358.536	1	30358.536	172.404
	PPES-R	39.151	1	39.151	14.462
Education	PrAS	327.677	1	327.677	1.861
	PPES-R	1.519	1	1.519	.561
Gestation	PrAS	1.664	1	1.664	.009
	PPES-R	.084	1	.084	.031
Parity	PrAS	1272.650	1	1272.650	7.227
	PPES-R	5.637	1	5.637	2.082
AgeGroup	PrAS	30.744	1	30.744	.175
	PPES-R	3.996	1	3.996	1.476
Parity * AgeGroup	PrAS	17.599	1	17.599	.100
	PPES-R	1.689	1	1.689	.624
Error	PrAS	61455.127	349	176.089	
	PPES-R	944.796	349	2.707	
Total	PrAS	1506244.000	356		
	PPES-R	25163.046	356		
Corrected Total	PrAS	94287.820	355		
	PPES-R	992.819	355		

Table S2. MANOVA: PrAS subscales and PPES-R subscales

Tests of Between-Subjects Effects

Source	Dependent Variable	Type III Sum of Squares	df	Mean Square	F	Sig.	Partial Eta Squared	Noncent. Parameter	Observed Power[l]
Corrected Model	PrASChildCon	27.028[a]	6	4.505	7.360	.000	.114	44.157	1.000
	PrASBodyI	89.699[b]	6	14.950	24.736	.000	.302	148.414	1.000
	PrASAttitBirth	32.497[c]	6	5.416	8.620	.000	.131	51.717	1.000
	PrASWorry	62.369[d]	6	10.395	46.488	.000	.448	278.929	1.000
	PrasBabyCon	34.821[e]	6	5.804	9.579	.000	.144	57.473	1.000
	PrasAccept	6.691[f]	6	1.115	4.710	.000	.076	28.261	.989
	PrasAvoid	3.172[g]	6	.529	1.162	.326	.020	6.974	.458
	PrASMed	46.745[h]	6	7.791	10.617	.000	.157	63.702	1.000
	PPES-R_Practical	45.909[i]	6	7.652	2.311	.034	.039	13.866	.800
	PPES-R_Emotional	44.996[j]	6	7.499	2.742	.013	.046	16.449	.873
	PPES-R_Understand	25.209[k]	6	4.202	1.455	.193	.025	8.728	.565
Intercept	PrASChildCon	9.432	1	9.432	15.409	.000	.043	15.409	.975
	PrASBodyI	.028	1	.028	.046	.830	.000	.046	.055
	PrASAttitBirth	36.950	1	36.950	58.804	.000	.146	58.804	1.000
	PrASWorry	.001	1	.001	.003	.959	.000	.003	.050
	PrasBabyCon	8.851	1	8.851	14.609	.000	.041	14.609	.968
	PrasAccept	7.135	1	7.135	30.138	.000	.081	30.138	1.000
	PrasAvoid	15.238	1	15.238	33.500	.000	.089	33.500	1.000
	PrASMed	7.994	1	7.994	10.894	.001	.031	10.894	.908
	PPES-R_Practical	920.013	1	920.013	277.883	.000	.448	277.883	1.000
	PPES-R_Emotional	1171.013	1	1171.013	428.081	.000	.555	428.081	1.000
	PPES-R_Understand	842.181	1	842.181	291.596	.000	.459	291.596	1.000
IPIP	PrASChildCon	19.217	1	19.217	31.396	.000	.084	31.396	1.000
	PrASBodyI	79.343	1	79.343	131.278	.000	.277	131.278	1.000
	PrASAttitBirth	16.284	1	16.284	25.915	.000	.070	25.915	.999
	PrASWorry	56.779	1	56.779	253.929	.000	.425	253.929	1.000
	PrasBabyCon	26.435	1	26.435	43.631	.000	.113	43.631	1.000
	PrasAccept	4.266	1	4.266	18.018	.000	.050	18.018	.988
	PrasAvoid	1.739	1	1.739	3.823	.051	.011	3.823	.496
	PrASMed	35.534	1	35.534	48.423	.000	.124	48.423	1.000

	PPES-R_Practical	24.279	1	24.279	7.333	.007	.021	7.333	.770
	PPES-R_Emotional	40.307	1	40.307	14.735	.000	.041	14.735	.969
	PPES-R_Understand	17.658	1	17.658	6.114	.014	.018	6.114	.693
Education	PrASChildCon	1.700	1	1.700	2.778	.096	.008	2.778	.383
	PrASBodyl	.172	1	.172	.284	.594	.001	.284	.083
	PrASAttitBirth	.123	1	.123	.196	.658	.001	.196	.073
	PrASWorry	.595	1	.595	2.661	.104	.008	2.661	.370
	PrasBabyCon	.582	1	.582	.960	.328	.003	.960	.165
	PrasAccept	1.015	1	1.015	4.289	.039	.012	4.289	.542
	PrasAvoid	.038	1	.038	.084	.772	.000	.084	.060
	PrASMed	.004	1	.004	.006	.938	.000	.006	.051
	PPES-R_Practical	1.858	1	1.858	.561	.454	.002	.561	.116
	PPES-R_Emotional	2.701	1	2.701	.987	.321	.003	.987	.168
	PPES-R_Understand	.879	1	.879	.304	.581	.001	.304	.085
Gestation	PrASChildCon	.034	1	.034	.055	.814	.000	.055	.056
	PrASBodyl	5.862	1	5.862	9.699	.002	.027	9.699	.874
	PrASAttitBirth	3.422	1	3.422	5.445	.020	.016	5.445	.643
	PrASWorry	.000	1	.000	.001	.980	.000	.001	.050
	PrasBabyCon	3.969	1	3.969	6.550	.011	.019	6.550	.723
	PrasAccept	.441	1	.441	1.864	.173	.005	1.864	.275
	PrasAvoid	.004	1	.004	.009	.922	.000	.009	.051
	PrASMed	2.347	1	2.347	3.198	.075	.009	3.198	.430
	PPES-R_Practical	.034	1	.034	.010	.920	.000	.010	.051
	PPES-R_Emotional	1.014	1	1.014	.371	.543	.001	.371	.093
	PPES-R_Understand	3.187	1	3.187	1.103	.294	.003	1.103	.182
Parity	PrASChildCon	5.996	1	5.996	9.795	.002	.028	9.795	.877
	PrASBodyl	.131	1	.131	.216	.642	.001	.216	.075
	PrASAttitBirth	10.805	1	10.805	17.195	.000	.048	17.195	.985
	PrASWorry	1.068	1	1.068	4.777	.030	.014	4.777	.587
	PrasBabyCon	2.118	1	2.118	3.495	.062	.010	3.495	.462
	PrasAccept	1.001	1	1.001	4.230	.040	.012	4.230	.536
	PrasAvoid	.071	1	.071	.157	.693	.000	.157	.068
	PrASMed	.038	1	.038	.051	.821	.000	.051	.056
	PPES-R_Practical	18.155	1	18.155	5.483	.020	.016	5.483	.646
	PPES-R_Emotional	.009	1	.009	.003	.954	.000	.003	.050
	PPES-R_Understand	2.060	1	2.060	.713	.399	.002	.713	.134
AgeGroup	PrASChildCon	.017	1	.017	.027	.868	.000	.027	.053
	PrASBodyl	.004	1	.004	.007	.932	.000	.007	.051
	PrASAttitBirth	.753	1	.753	1.199	.274	.003	1.199	.194
	PrASWorry	.088	1	.088	.394	.531	.001	.394	.096

	PrasBabyCon	1.780	1	1.780	2.938	.087	.008	2.938	.401
	PrasAccept	.014	1	.014	.060	.806	.000	.060	.057
	PrasAvoid	.307	1	.307	.674	.412	.002	.674	.130
	PrASMed	.297	1	.297	.405	.525	.001	.405	.097
	PPES-R_Practical	3.830	1	3.830	1.157	.283	.003	1.157	.189
	PPES-R_Emotional	1.820	1	1.820	.665	.415	.002	.665	.129
	PPES-R_Understand	3.977	1	3.977	1.377	.241	.004	1.377	.216
Parity * AgeGroup	PrASChildCon	.001	1	.001	.001	.971	.000	.001	.050
	PrASBodyl	.432	1	.432	.714	.399	.002	.714	.134
	PrASAttitBirth	.082	1	.082	.131	.718	.000	.131	.065
	PrASWorry	.069	1	.069	.308	.579	.001	.308	.086
	PrasBabyCon	.546	1	.546	.902	.343	.003	.902	.157
	PrasAccept	.322	1	.322	1.361	.244	.004	1.361	.214
	PrasAvoid	.037	1	.037	.081	.776	.000	.081	.059
	PrASMed	.003	1	.003	.004	.948	.000	.004	.050
	PPES-R_Practical	.231	1	.231	.070	.792	.000	.070	.058
	PPES-R_Emotional	.336	1	.336	.123	.726	.000	.123	.064
	PPES-R_Understand	.022	1	.022	.008	.931	.000	.008	.051
Error	PrASChildCon	209.946	343	.612					
	PrASBodyl	207.304	343	.604					
	PrASAttitBirth	215.527	343	.628					
	PrASWorry	76.695	343	.224					
	PrasBabyCon	207.814	343	.606					
	PrasAccept	81.206	343	.237					
	PrasAvoid	156.017	343	.455					
	PrASMed	251.698	343	.734					
	PPES-R_Practical	1135.603	343	3.311					
	PPES-R_Emotional	938.274	343	2.735					
	PPES-R_Understand	990.645	343	2.888					
Total	PrASChildCon	1857.000	350						
	PrASBodyl	2137.480	350						
	PrASAttitBirth	2507.778	350						
	PrASWorry	1250.972	350						
	PrasBabyCon	1538.000	350						
	PrasAccept	682.000	350						
	PrasAvoid	906.222	350						
	PrASMed	1695.778	350						
	PPES-R_Practical	23582.967	350						
	PPES-R_Emotional	29463.420	350						
	PPES-R_Understand	21277.480	350						

Corrected Total	PrASChildCon	236.974	349						
	PrASBodyI	297.004	349						
	PrASAttitBirth	248.024	349						
	PrASWorry	139.064	349						
	PrasBabyCon	242.635	349						
	PrasAccept	87.897	349						
	PrasAvoid	159.189	349						
	PrASMed	298.443	349						
	PPES-R_Practical	1181.512	349						
	PPES-R_Emotional	983.271	349						
	PPES-R_Understand	1015.854	349						

a. R Squared = .114 (Adjusted R Squared = .099)

b. R Squared = .302 (Adjusted R Squared = .290)

c. R Squared = .131 (Adjusted R Squared = .116)

d. R Squared = .448 (Adjusted R Squared = .439)

e. R Squared = .144 (Adjusted R Squared = .129)

f. R Squared = .076 (Adjusted R Squared = .060)

g. R Squared = .020 (Adjusted R Squared = .003)

h. R Squared = .157 (Adjusted R Squared = .142)

i. R Squared = .039 (Adjusted R Squared = .022)

j. R Squared = .046 (Adjusted R Squared = .029)

k. R Squared = .025 (Adjusted R Squared = .008)

l. Computed using alpha = .05
